# A Novel Modelling Methodology Which Predicts the Structural Behaviour of Vertebral Bodies under Axial Impact Loading: A Finite Element and DIC Study

**DOI:** 10.3390/ma13194262

**Published:** 2020-09-24

**Authors:** Bruno Agostinho Hernandez, Harinderjit Singh Gill, Sabina Gheduzzi

**Affiliations:** Department of Mechanical Engineering, University of Bath, Bath BA2 7AY, UK; bah33@bath.ac.uk (B.A.H.); rg433@bath.ac.uk (H.S.G.)

**Keywords:** vertebral body, finite element model, DIC, stiffness, dynamic loading, impact

## Abstract

Cervical spine injuries (CSIs) arising from collisions are uncommon in contact sports, such as rugby union, but their consequences can be devastating. Several FE modelling approaches are available in the literature, but a fully calibrated and validated FE modelling framework for cervical spines under compressive dynamic-impact loading is still lacking and material properties are not adequately calibrated for such events. This study aimed to develop and validate a methodology for specimen-specific FE modelling of vertebral bodies under impact loading. Thirty-five (n = 35) individual vertebral bodies (VBs) were dissected from porcine spine segments, potted in bone cement and μCT scanned. A speckle pattern was applied to the anterior faces of the bones to allow digital image correlation (DIC), which monitored the surface displacements. Twenty-seven (n = 27) VBs were quasi-statically compressively tested to a load up to 10 kN from the cranial side. Specimen-specific FE models were developed for fourteen (n = 14) of the samples in this group. The material properties were optimised based on the experimental load-displacement data using a specimen-specific factor ($k_{GS_{static}}$) to calibrate a density to Young’s modulus relationship. The average calibration factor arising from this group was calculated (${\bar{K}}_{GS_{static}}$) and applied to a control group of thirteen (n = 13) samples. The resulting VB stiffnesses was compared to experimental findings. The final eight (n = 8) VBs were subjected to an impact load applied via a falling mass of 7.4 kg at a velocity of 3.1 m s^−1^. Surface displacements and strains were acquired from the anterior VB surface via DIC, and the impact load was monitored with two load cells. Specimen-specific FE models were created for this dynamic group and material properties were assigned again based on the density–Young’s modulus relationship previously validated for static experiments, supplemented with an additional factor ($K_{GS_{dynamic}}$). The optimised conversion factor for quasi-static loading, ${\bar{K}}_{GS_{static}}$, had an average of 0.033. Using this factor, the validation models presented an average numerical stiffness value 3.72% greater than the experimental one. From the dynamic loading experiments, the value for $K_{GS_{dynamic}}$ was found to be 0.14, 4.2 times greater than ${\bar{K}}_{GS_{static}}$. The average numerical stiffness was 2.3% greater than in the experiments. Almost all models presented similar stiffness variations and regions of maximum displacement to those observed via DIC. The developed FE modelling methodology allowed the creation of models which predicted both static and dynamic behaviour of VBs. Deformation patterns on the VB surfaces were acquired from the FE models and compared to DIC data, achieving high agreement. This methodology is now validated to be fully applied to create whole cervical spine models to simulate axial impact scenarios replicating rugby collision events.

## 1. Introduction

Cervical spine injuries (CSIs) are one of the most threatening injuries to the spine [1,2,3,4]. CSIs can lead to paraplegia, tetraplegia or even death. Therefore, understanding how such injuries occur is crucial in the development of new treatments and injury-specific prevention plans and to increase the long-term well-being of CSI patients [5].

The majority of CSIs occur due to impacts to the head, usually in road traffic accidents, falls, diving and sports events [1]. Research focussed on CSI arising from contact sports has gained popularity [6,7,8,9,10,11], as understanding the injury mechanism could lead to better rule making by the sporting authorities, and hopefully, result in a decrease in injury occurrence. This research field, however, is characterised by significant challenges, including the fact that the instantaneous nature of the injury makes its assessment difficult with traditional techniques. This is certainly one of the challenges faced when studying CSIs arising from rugby, wherein views can easily be obstructed by other players. Hence experimental and in vivo studies have been used as study surrogates; however, there still is no consensus as to the mechanisms ultimately leading to injury [9,12].

Numerical methods have been increasingly applied to predict CSI occurrence in a range of scenarios and to analyse situations that cannot be replicated in vitro [13]. Among these, the finite element (FE) method is widely used [14,15,16].

One of the main challenges when creating cervical spine models is to appropriately determine the material properties of the bone components. Like many other biological materials, bone displays time-dependent (viscoelastic) behaviour [17,18,19,20,21,22]. In other words, its mechanical properties are dependent upon the rate of application of the load. This well-known fact is seldom accounted for when developing dynamic FE models. The majority of the dynamic FE studies either set the vertebral bodies (VBs) as rigid bodies [23,24,25], or use quasi-static bone properties [26,27,28,29], or use non-calibrated elastic-plastic material laws with a non-calibrated hardening parameter [26,30,31], or arbitrarily increase Young’s modulus from quasi-static regime to match the rise in stiffness [28,32,33]. Appropriate material properties, however, are essential for the accuracy of finite element models. For example, Kazarian [20] and others [33,34,35] have shown that buckling deformation and burst and wedge fractures frequently occur at high loading rates; thus, setting vertebral bodies as rigid entities does not replicate these conditions. Besides, this simplification results in an overloading of the IVD and alters the kinematic response of the spine.

Due to the popularity of this method, many different spine modelling procedures are described in the literature. These vary widely in terms of geometry description, boundary conditions and load application, rendering direct comparisons between different studies a difficult task [15,16,36,37]. A calibrated and validated FE modelling framework, with material properties appropriate to the loading rate, is thus necessary in order to standardise the FE modelling procedures and to allow a more realistic representation of bone behaviour in such extreme loading scenarios.

Therefore, this study aimed to develop a calibrated and validated finite element modelling methodology to create specimen-specific vertebral body models calibrated to impact loading scenarios. This methodology represents a fundamental step to allow the development of realistic whole spine models to investigate cervical spine injuries.

## 2. Materials and Methods

Porcine vertebral bodies were subjected to two experimental testing protocols: quasi-static calibration and validation, and dynamic compression. The first one aimed to develop, calibrate and validate a finite element modelling methodology for quasi-static loading of vertebral bodies. The second aimed to adapt the previously validated quasi-static FE modelling methodology to allow the investigation of dynamic impacts typical of sports collisions, such as those in rugby union, on the behaviour of vertebral bodies.

### 2.1. Specimen Preparation

Thirteen porcine C2–T1 spinal segments, originating from animals aged between 8 and 12 months at the time of slaughter, were purchased from a local abattoir (Langford Abattoir, Langford, Bristol). Thirty five (n = 35) individual vertebral bodies (VBs) were dissected from the spinal segments and all soft tissue, pedicles and processes were removed. The superior and inferior end plates of each VB were potted in custom cylindrical polytetrafluoroethylene (PTFE) moulds using bone cement (Simplex, Stryker Corporation, Kalamazoo, MI, USA). USA). The samples were divided into three groups (Table 1): the first group was used for quasi-static calibration of the finite element modelling methodology, the second was used to validate said methodology and the third was used to validate the dynamic compression simulations.

### 2.2. μCT Imaging

The VB samples were μCT scanned alongside phantom discs using a Nikon XTH225ST Micro-CT Scanner Unit (Nikon Metrology Inc., Brighton, MI, USA) equipped with a tungsten filament. Scanning was carried out in circular CT mode with peak voltage 142 kV, current of 137 μA, slice spacing and slice reconstruction of 0.2 mm, pixel dimensions 121.8 μm× 121.8 μm, with a float data type of 32 bits per element. Two images per projection were acquired and no filter was used. Images were reconstructed from 1800 projections using CT Pro-3D software (Nikon Metrology Inc., Brighton, MI, USA).

### 2.3. Digital Image Correlation

In order to allow digital image correlation (DIC) measurements to be performed, a black speckle pattern was applied over a coat of white primer on the anterior aspect of each VB. Care was taken to ensure a homogeneous dot pattern, larger than 3 pixels and characterised by density ratio between white and black ranging from 30% to 50%, was achieved [38].

Two different DIC set-ups were used; in the case of quasi-static experiments DIC was acquired through a single digital camera (GigE DFK 23GP01, The Imaging Source Europe GmbH, Überseetor Bremen, Germany), with image resolution of 72 dpi and window size of 2592 × 1944. Care was taken to ensure the camera lens was parallel and level with the anterior aspect of the VB. Images were acquired by a custom MatLab algorithm (MathWorks, Natick, MA, USA) at a rate of 1 image every 5 s. Ncorr V2.1 [39] was used for post-processing and analysis conducted using a radius of 30 pixels and spacing of 5 pixels. DIC measurements during dynamic experiments were performed by means of two high-speed cameras (Fastcam SA3 Master and Slave, Photron Europe Ltd., High Wycombe, UK). In this case the image acquisition rate was set at 4000 frames per second, with a resolution of 96 dpi and window size of 512 × 762 pixels. Vic-3D (Correlated Solutions, Irmo, SC, USA) was used for post-processing and analysis conducted with radius of 30 pixels and spacing of 5 pixels [40].

### 2.4. Mechanical Testing

Each VB in the quasi-static group was subjected to a compressive ramp to a maximum load of 10 kN, applied at a rate of 1 kN min^−1^. Load was applied at the cranial side of each VB via a 35 mm ball bearing mounted on the crosshead of a 30 kN material testing machine (Instron 5967, Instron, High Wycombe, UK) to prevent transmission of unwanted shear forces to the specimen. A steel plate was inserted between the ball bearing and the superior cement end cap to allow for uniform load application and to minimise the insurgence of localised deformation at the point of application of the load. The position of the point of load application was determined prior to testing and located by measuring its distance from predefined landmarks.

The dynamic experiment samples were mounted on an impact cage equipped with two load cells, one placed at the cranial and one at the caudal end of the specimen [41]; in this set-up the caudal end of the VB was fully constrained and only vertical axial displacement was allowed at the cranial side. A mass of 7.4 kg falling from a height of 0.5 m impacted the cranial loading platform. Under these conditions the mass was accelerated to a maximum velocity of 3.1 m s^−1^, hence delivering a blow of 35.5 J, thereby resulting in energy levels similar to those reported for collisions arising from rugby [41,42,43,44,45].

Experimental displacement data were acquired via DIC from the majority of the anterior surface of the sample but only a defined ROI was used to calculate vertebral stiffness. Load from the testing machine, for the quasi-static experiment, and from the bottom load cell, for the dynamic experiment, was used and combined for DIC displacements to plot load-displacement curves for all samples.

### 2.5. Finite Element Geometrical Modelling

The μCT images of each specimen, comprising VB, cartilage remnants and bone cement end caps, were imported into ScanIP software (v2017-18, Simpleware Synopsys, Mountain View, CA, USA) and downsized from a voxel size of 0.12 mm to a voxel size of 0.40 mm. At this resolution sufficient detail was retained to allow accurate models to be created while significantly reducing the number of slices, from approximately 1600 to 400.

VB bone modelling included thresholding, flood filling, interpolation, painting and filtering to create specimen fidelic geometries, while bone cement specimen-holder caps and cartilage were modelled using thresholding and Boolean tools to obtain a perfect contact interface between parts. Cartilage was only included in the model when its thickness exceeded three pixels to prevent model distortion [46]. The steel plate used in the experiments to apply the load was reconstructed based on its physical dimensions and positioned over the cranial cement cap. Its alignment with respect to the VB was determined based on physical measurements acquired during the experimental set-up. Once the geometrical modelling step was completed, the resulting geometries were re-imported into the 0.12 mm pixel size file to exploit the high-resolution voxel size while setting material properties.

A mesh sensitivity test was conducted, resulting in an optimum element size of 1 ${\mathrm{mm}}^{3}$. The final geometrical models were downscaled again, but from 0.12 mm to 1 mm voxel size and each voxel was converted into finite elements using the meshing tool within the Simpleware software package. Linear 1 ${\mathrm{mm}}^{3}$ hexahedron elements were applied to the interior volume of the geometry, with the principal axis aligned in the caudal-cranial direction. Linear tetrahedron elements were used to smooth outer surfaces [13,47,48]. The final geometry was then exported to ANSYS Mechanical APDL FE software (v18.2, ANSYS Inc., Canonsburg, PA, USA).

### 2.6. Finite Element Boundary Conditions and Load Application

**Figure 1:**
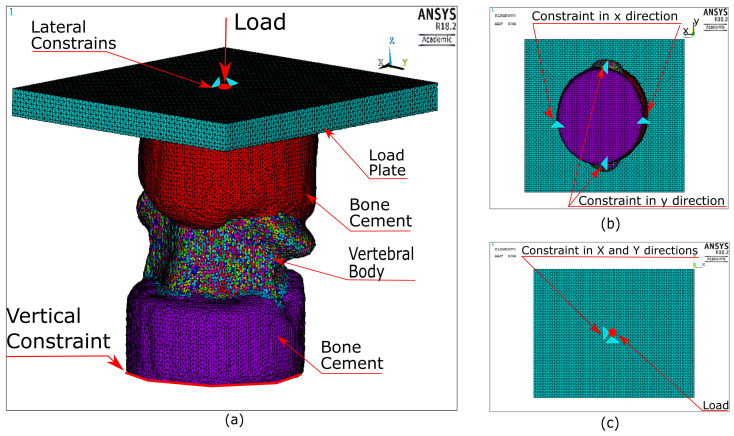
Boundary conditions applied into vertebral body FE models. (**a**) Perspective view of a typical VB FE model and its BCs. (**b**) Caudal view depicting the constrains in movement. (**c**) Cranial view illustrating the loading point and its constraints.

The quasi-static simulation boundary conditions constrained the caudal bone cement cap in the caudal-cranial direction (*z*-axis, Figure 1a), additionally four external nodes on the cap surface were constrained to prevent rotations and translations of the sample (Figure 1b). The node closest to the experimental point of application of the load was identified at the top surface of the steel plate based on the measurements of its position acquired prior to each experiment. This node was constrained in the posterior-anterior and medio-lateral directions (*x* and *y*-axes, respectively, Figure 1a,b) to represent the contact restriction caused by the ball bearing (Figure 1c). A compressive loading ramp, ranging from 0 to 10 kN in a pseudo-time of 1 s, was applied to this node. The analysis type was set as quasi-static and implicit. No contact was applied and all bodies were considered bonded.

In the dynamic simulations the caudal specimen holder was fully constrained while the cranial specimen holder was only allowed to move vertically. The experimental load acquired from the cranial load cell (cranial load) was applied vertically via the rigid steel placed on top of the superior cement cap. No contact was used and all parts were considered bonded. The solution was run using ANSYS inbuilt LS-DYNA solver (v4.2, Livermore Software Technology Corporation (LSTC), Livermore, CA, USA) and an explicit analysis was defined.

Numerical displacement data was acquired from the vertebral body anterior surface nodes on a similar ROI defined for the experimental data. This ROI was defined based on visual landmarks on bone cement. Load data were acquired from the bottom nodes with vertical constraints for the quasi-static models. For the dynamic models, experimental load data were used, as will be explained in later sections. Load-displacement curves were then created and stiffness was measured from them.

### 2.7. Finite Element Material Properties

The material properties of bone cement and the steel plate were defined as homogeneous and isotropic, with the first being obtained from preliminary experiments, and the latter from the literature; cartilage was set as an homogeneous and hyperelastic material (Table 2). A transversally linear isotropic, element-based material model, having the characteristics outlined in Equation (1), was chosen for the VBs [49,50,51,52,53,54]:

(1) $$\begin{array}{c} \begin{array}{cc} E_{xx} & =0.333\;\cdot \;E_{zz}\\ E_{yy} & =0.333\;\cdot \;E_{zz}\\ G_{xy} & =0.121\;\cdot \;E_{zz}\\ G_{xz} & =0.157\;\cdot \;E_{zz}\\ G_{yz} & =0.157\;\cdot \;E_{zz}\\ \nu_{xy} & =0.381\\ \nu_{xz} & =0.104\\ \nu_{yz} & =0.104 \end{array} \end{array}$$

where $E_{zz}$ is Young’s modulus in the principal direction, in this case caudal-cranial, while $E_{yy}$ and $E_{xx}$ are Young’s moduli in the posterior-anterior and medial-lateral directions. $G_{xy}$, $G_{xz}$ and $G_{yz}$ are the shear moduli in the planes perpendicular to the caudal-cranial, posterior-anterior and medial-lateral directions; $\nu_{xy}$, $\nu_{xz}$ and $\nu_{yz}$ are the Poisson’s ratios in the directions perpendicular to caudal-cranial, posterior-anterior and medial-lateral, respectively.

The magnitude of $E_{zz}$ was assigned based on its relationships with apparent density developed for human VBs [55], scaled by an empirical, specimen specific, factor ${k_{GS}}_{static}$:

(2) $$E_{zz}={k_{GS}}_{static}\left(-0.00347+0.323\;\cdot \;\rho_{app}\right)$$

where $\rho_{app}$ is the pixel apparent density, in $kg\;m^{-3}$, and the scaling factor, ${k_{GS}}_{static}$, was introduced to account for the difference in density between human and porcine samples. The value of $\rho_{app}$ was derived from the pixel grey-scale value via the two phantoms μCT scanned alongside each sample. Between 40 and 60 different material properties were thus generated for each VB, as recommended [40,56,57]. The purpose of the 14 quasi-static calibration models was to identify optimal values for ${k_{GS}}_{static}$ that minimised the differences between numerical predictions and experimental measurements of specimen stiffness.

The transversally isotropic model of Equation (1) was applied to the 13 quasi-static validation models, except this time, a single average scaling factor ${{\bar{K}}_{GS}}_{static}$, calculated from the 14 specimen specific scaling factors ${k_{GS}}_{static}$, was substituted in Equation (2). The effect of adopting an average scaling factor (${{\bar{K}}_{GS}}_{static}$) on numerical stiffness predictions was evaluated against experimental value in the validation group.

A number of assumptions were made when assigning material properties in the dynamic simulation models; they find their basis in the increase in stiffness response exhibited by viscoelastic materials when subjected to loading at increasing rates. We postulated that a relationship between Young’s modulus and density of the type presumed by Equation (2) would hold true for any loading rate; however, a new calibration parameter would be required to account for the effect of the increased loading rate. With this assumption the new calibration factor, ${K_{GS}}_{dynamic}$, is related to ${{\bar{K}}_{GS}}_{static}$ via a constant of proportionality α (Equation (3)):

(3) $$K_{GS_{dynamic}}=\alpha \;\cdot \;{{\bar{K}}_{GS}}_{static}\;\;\;with\;\;\alpha >1$$

With this assumption the transversally linear isotropic material could also be applied to the dynamic simulation group, and an iterative approach was carried out to quantify α so the following conditions were met: (a) The differences between average experimental and predicted stiffness (among all models) should be less than 5%. (b) The Bland–Altman plot should be centred around (or close to) zero. (c) The non-parametric Mann–Whitney U-test should return no significant differences.

A further point to highlight with respect to the dynamic simulations is that bone cement and polyoxymethylene specimen holders were defined as rigid bodies [60]. As the rigid body constraints were set at the centres of mass of the objects, the reaction forces from the caudal constraints were not available for these simulations; therefore, experimental load data from the bottom load cell were used to produce load-displacement curves.

## 3. Results

A small representative sample of the results is presented within the main body of this paper. The remaining set of results is presented in the Supplementary Material. The static calibration samples lead to specimen specific calibration factors, $k_{GS_{static}}$, ranging from 0.021 to 0.048, resulting in an average calibration factor, ${{\bar{K}}_{GS}}_{static}$, of 0.033 ± 0.009.

**Figure 2:**
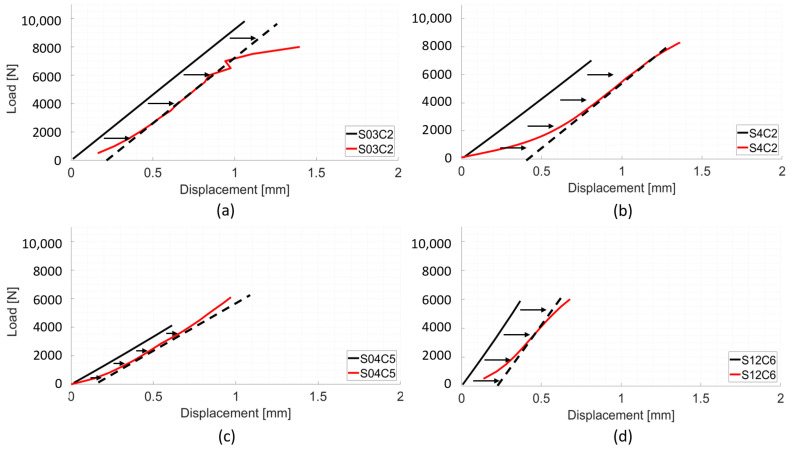
Representative sample of load-displacement curves. Red curves are DIC/experimental results. Black curves are numerical results. Stiffness was calculated from 3 kN–5 kN. (**a**) Calibration model of spine 03, sample C2; (**b**) Calibration model of spine 04, sample C2. (**c**) Validation model of spine 04, sample C5. (**d**) Validation model of spine 12, sample C6.

With the individually optimised ${k_{GS}}_{static}$ values, the average difference between experimental and numerical stiffness values in the quasi-static calibration group was less than 1%, 8909 ± 2156 $N\;{\mathrm{mm}}^{-1}$ and 8936 ± 2121 $N\;{\mathrm{mm}}^{-1}$, respectively (Figure 2a,b). Experimental and numerical VB stiffness value distributions were compared using the non-parametric Mann–Whitney U-test and no statistical significant difference between the groups was found (*p* > 0.05); both were characterised by a median value of about 8600 N ${\mathrm{mm}}^{-1}$.

**Figure 3:**
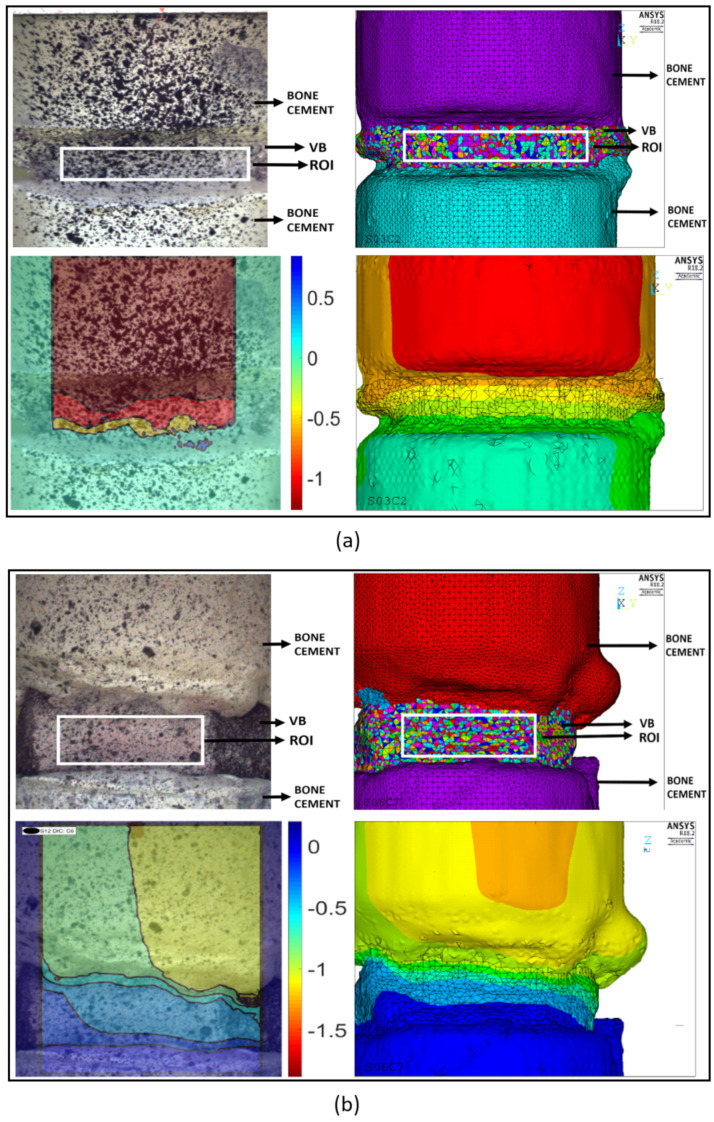
Representative sample of experimental and numerical contour plot results from quasi-static loading experiment. The load magnitude is 5 kN. Top left is the experimental-DIC data: DIC was acquired from the majority of the anterior surface of the sample but only the defined ROI (white square) was used to calculate vertebral stiffness. Top right is the similar ROI defined in the FE model. Bottom left is the DIC vertical displacement contour plot, in mm, adjusted to toe region width. Bottom right is FE vertical displacement contour plot, in mm. (**a**) Spine 03, sample C2 calibration results. (**b**) Spine 12, sample C6 validation results.

The majority of the models in the quasi-static calibration group presented displacement contour patterns for the vertebral body similar to those seen experimentally, with maximum displacement values located at the junction between the cranial bone cement cap and the vertebral body; the same pattern is shown by DIC data (Figure 3a). In general, FE and DIC displacement patterns were, in terms of magnitude and distribution, comparable.

**Figure 4:**
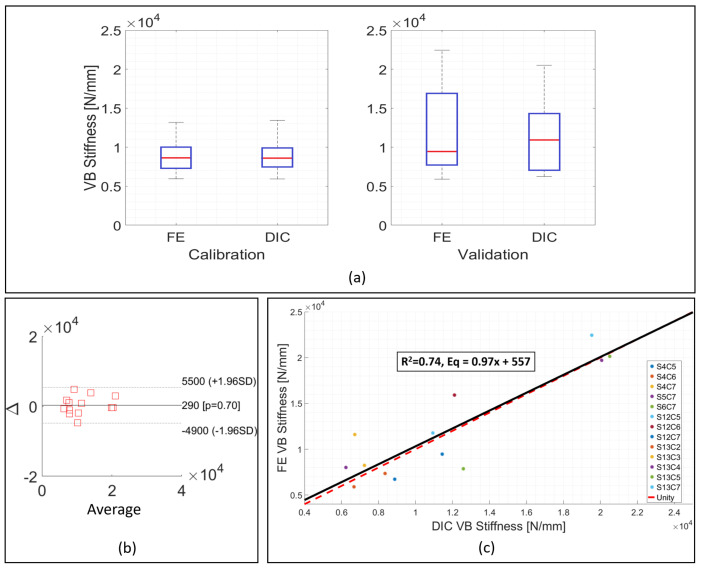
Experimental and numerical results for the validation models from quasi-static loading experiment. (**a**) Comparison between experimental and numerical stiffness variability in the calibration and validation phases. (**b**) Bland–Altman plot comparing experimental and numerical stiffness. Δ is the difference between experimental and numerical stiffness. Average is the average between experimental and numerical stiffness. (**c**) Correlation between experimental and numerical stiffness. $R^{2}$ = 0.74 for a relationship of 0.95. Dashed red line is the unit line for comparison.

For the whole of the validation set, the specimen specific individual factor, ${k_{GS}}_{static}$ in Equation (2) was substituted for the average calibration factor, ${{\bar{K}}_{GS}}_{static}$ = 0.033. This led to an average numerical stiffness value for the 13 validation samples of 12,007 ± 5858 $N\;{\mathrm{mm}}^{-1}$, 3.7% greater than the average experimental stiffness, 11,577 ± 5280 $N\;{\mathrm{mm}}^{-1}$. The differences for the majority of the samples ranged from 1.8% to 37.6% (Figure 2c,d). The average experimental stiffness for this group was higher compared to the calibration group, from 8936 ± 2121 $N\;{\mathrm{mm}}^{-1}$ to 11,577 ± 5280 $N\;{\mathrm{mm}}^{-1}$ (Figure 4a) and this was reflected by the predicted stiffness, which was also higher than in the calibration set, 12,007 ± 5858 $N\;{\mathrm{mm}}^{-1}$ compared to 8909 ± 2156 $N\;{\mathrm{mm}}^{-1}$.

Similarly to what observed was in the calibration set, the majority of the models presented similar ranges of values on the contour plots as obtained via DIC (Figure 3b). The non-parametric Mann Whitney U-test indicated no statistically significant difference between the numerical and experimental stiffness for the validation group (*p* > 0.05) (Figure 4a, right side), and the Bland–Altman plot showed the average difference between experimental and numerical stiffness to be around zero (Figure 4b). The correlation between experimental and numerical stiffness results presented a $R^{2}=0.74$ and a Lin’s concordance correlation coefficient (CCC) of 0.88, for a relationship of 0.95 (Figure 4c).

The calibration procedures for the dynamic models resulted in an α factor, from Equation (3), of 4.3, giving a value of $K_{GS_{dynamic}}$ = 0.14. This value fulfilled all three requirements previously outlined.

**Figure 5:**
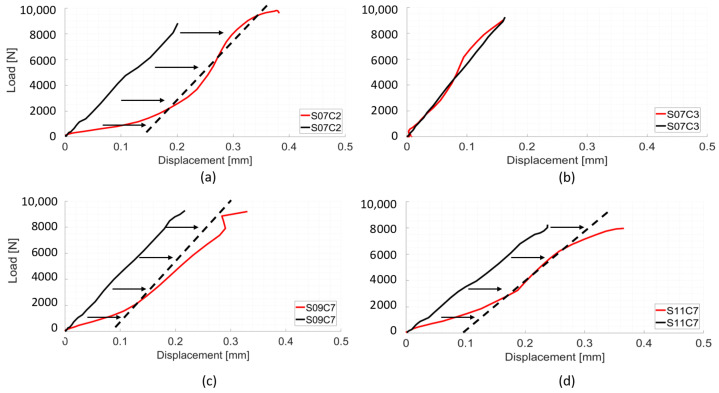
Representative sample of load-displacement curves from dynamic loading experiment. Red curve is the experimental/DIC result from the VB anterior surface. Black curve is the result from the VB anterior surface for models. (**a**) Spine 07, sample C2. (**b**) Spine 07, sample C3. (**c**) Spine 09, sample C7. (**d**) Spine 11, sample C7.

Dynamic experimental load-displacement curves presented similar features to those obtained from quasi-static experiments, displaying a toe region, followed by a linear section and a yield point, with maximum displacements ranging from 0.2 mm to 0.9 mm. Predicted load-displacement curves were almost completely linear (Figure 5).

Experimental stiffness values, estimated by a linear fit of the load-displacement curves in the region 3 kN to 5 kN, ranged from 26,035 N ${\mathrm{mm}}^{-1}$ to 75,236 N ${\mathrm{mm}}^{-1}$, with an average of 49,677 ± 16,850 $N\;{\mathrm{mm}}^{-1}$; predicted stiffness values ranged from 33,847 N ${\mathrm{mm}}^{-1}$ to 95,591 N ${\mathrm{mm}}^{-1}$, with averages of 50,794 ± 13,710 $N\;{\mathrm{mm}}^{-1}$. The difference between average experimental and numerical stiffness was 2.3%. The minimum and the maximum differences were 1.5% and 41.6%, respectively.

**Figure 6:**
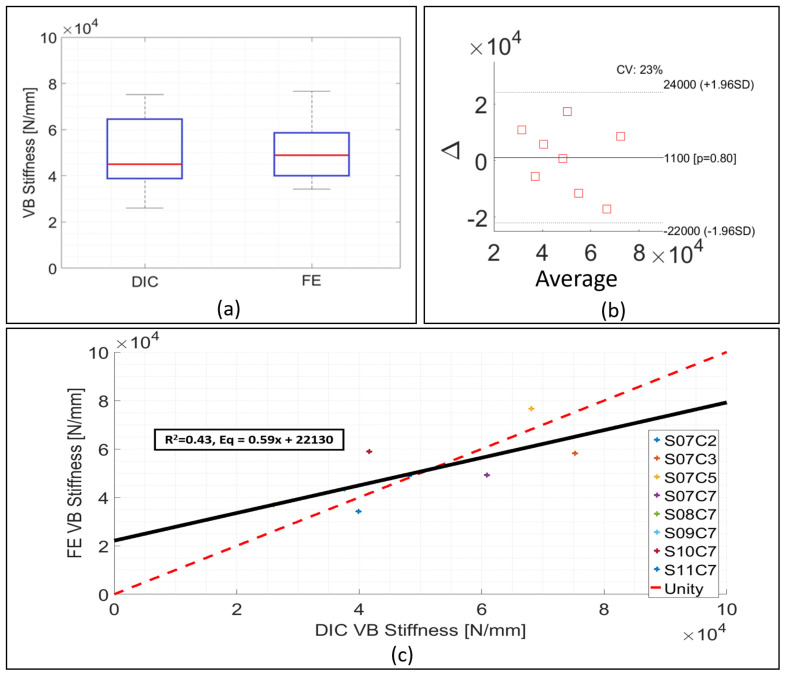
Experimental and numerical results for dynamic loading. (**a**) Bland–Altman plot comparing experimental and numerical stiffness for both BC models. Δ is the difference between experimental and numerical stiffness. Average is the average between experimental and numerical stiffness. (**b**) Box and whisker plot comparison between experimental and numerical stiffness. (**c**) Correlation plot comparing experimental and numerical stiffness values. $R^{2}$ = 0.43 for a relationship of 0.57.

The non-parametric Mann–Whitney U-test highlighted no statistically significant difference between numerical predictions and experimental estimates of stiffness (*p* > 0.05), Figure 6a, with the mean difference between experimental and numerical values being 1100 N ${\mathrm{mm}}^{-1}$, Figure 6b. The linear regression between experimental and numerical stiffness was characterised by a slope of 0.59, $R^{2}$ = 0.42, and a Lin’s concordance correlation coefficient (CCC) of 0.70 (Figure 6c).

**Figure 7:**
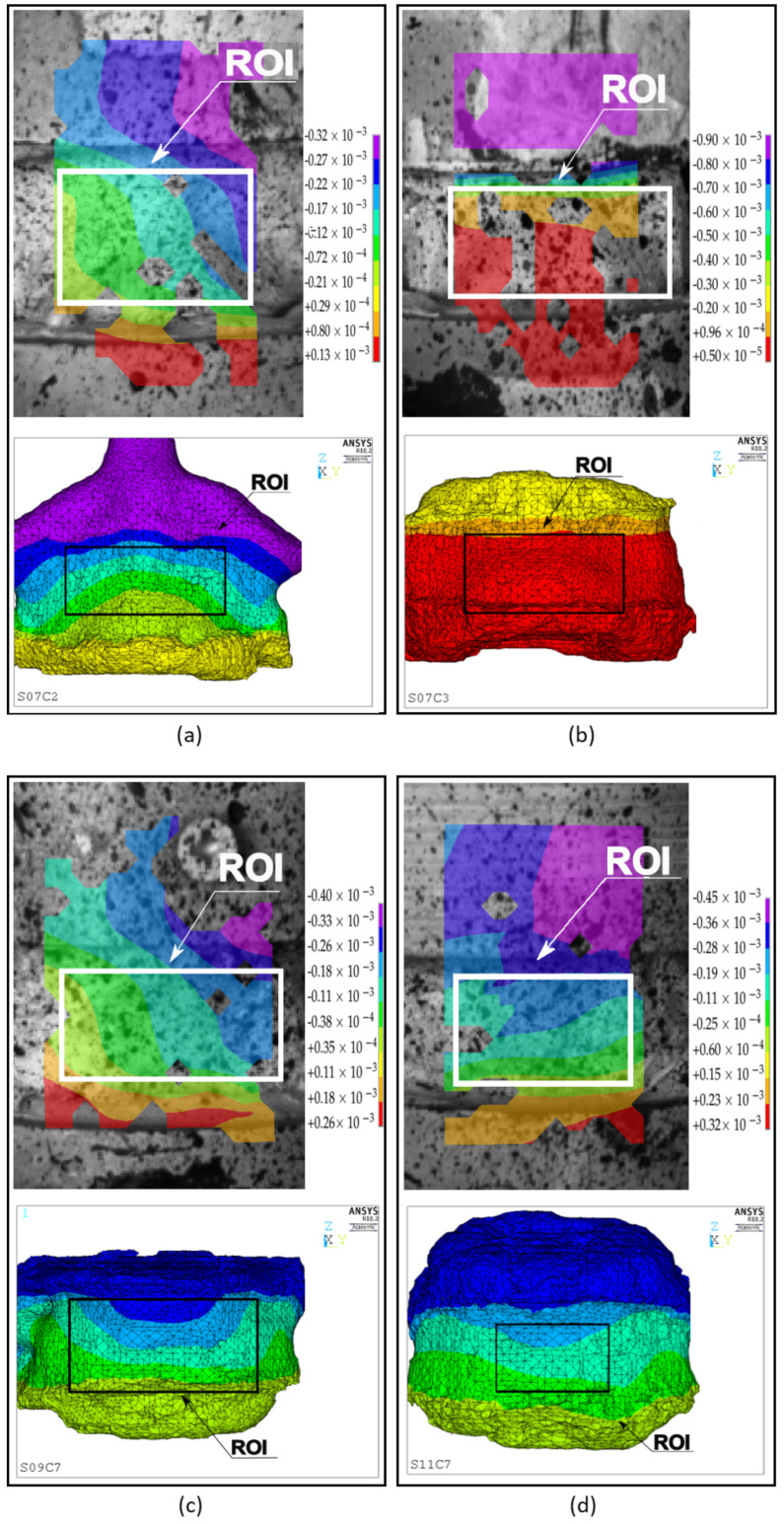
Representative sample of experimental and numerical contour plot results for dynamic loading. Top image shows the experimental/DIC results and bottom image shows the numerical results. The load magnitude is 5 kN. DIC and numerical vertical displacements, in m. (**a**) Spine 07, sample C2. (**b**) Spine 07, sample C3. (**c**) Spine 09, sample C7. (**d**) Spine 11, sample C7.

The majority of the models presented similar displacement distributions contours to those obtained with DIC, Figure 7. Maximum experimental displacement values ranged from 0.3 mm to 0.8 mm, while numerical displacement results ranged between 0.1 mm and 0.5 mm. Maximum displacement regions were located in the same areas, as shown by DIC: at the cranial junction between cement and vertebral body.

## 4. Discussion

Cervical spine injuries (CSIs) and severe spinal trauma are often related to fast events. As a result, there is great interest in replicating these events in the laboratory to quantify and to determine injury mechanisms and human tolerance to injury [1]. In vitro experiments in this area of research, however, are often time-consuming, expensive and specimen and loading scenario-dependent. Furthermore, the dynamic nature of such events makes it difficult to visualise the exact moment of injury.

The aim of this study was to develop a validated modelling methodology able to create specimen-specific finite element models of vertebral bodies calibrated for dynamic loading scenario. This framework could be applied to create whole spine models to evaluate impact-like events.

The first step towards devising such methodology is to appropriately set material properties. A validated linear equation [55] was chosen as the basis from which to set specimen-specific material properties based on the grey-scale of VB images. This equation was selected due to the reported high correlation between density and Young’s modulus and its recurrence in the literature [13,52,53,54].

A calibration factor, $k_{GS_{static}}$, was used to adjust the equation, originally derived from elderly human cadavers [55], to the samples utilised in this study, i.e., juvenile porcine VBs, on a specimen by specimen basis. This calibration was necessary to create a modelling methodology, in order for it to be used in other studies, allowing a direct comparison between studies. The multitudes of unvalidated and uncalibrated modelling methodologies and models available in the literature [37] make the comparisons difficult between studies, thereby rendering the drawing of broader conclusions a challenging task.

In this preliminary calibration phase, each sample was subjected to a quasi-static loading ramp and values obtained for experimental and numerical stiffness were compared, resulting in average values within 1% of each other, 8909 ± 2156 $N\;{\mathrm{mm}}^{-1}$ and 8936 ± 2121 $N\;{\mathrm{mm}}^{-1}$, respectively. In comparison with studies which used human lumbar VBs (the largest dataset in the literature), the values of stiffness obtained here were either similar [61,62,63,64,65] or smaller [53,66,67,68].

An average calibration factor, ${\bar{K}}_{GS_{static}}$ was derived from the specimen specific values of $k_{GS_{static}}$, and the effect of using this value in Equation 2 was evaluated on the 13 samples comprising the validation set. For the validation models, although some differences between numerical and experimental stiffness were around 30%, with one outlier being up to 73%, the Bland–Altman plot presented distributions of the differences between experimental and numerical around and close to zero (Figure 4b). Moreover, the relationship between experimental and numerical data was 0.95, and a high correlation was found, $R^{2}$ = 0.74. This represents an improvement compared to what was reported in the literature, with other studies achieving values in the order 0.50 to 0.72 [62,63,66,67]. The box and whisker plot further highlighted the similarities in the validation and calibration of samples, Figure 4a.

Compared to the calibration phase, the average experimental stiffness increased in the validation set, from around 8936 $N\;{\mathrm{mm}}^{-1}$ to 11,570 $N\;{\mathrm{mm}}^{-1}$. This was also seen in the FE models (Figure 4a) with average numerical stiffness for the calibration dataset being 8909 ± 2156 $N\;{\mathrm{mm}}^{-1}$ compared to 12,007 ± 5858 $N\;{\mathrm{mm}}^{-1}$ for the validation set. In other words, the FE models were able to represent what was seen experimentally, as the increase in the experimental stiffness from one phase to another was also reflected in the numerical results.

In order to quantify the agreement between experimental and numerical results, the Lin’s concordance correlation coefficient (CCC) was used [69]. The concordance correlation coefficient provides a measure of how well data points fit into a one-to-one relationship (x=y) as opposed to the correlation coefficient, which only provides an indication of how linear the regression line is [70], without assessing the nature of the relationship. The CCC of the fitting line between experimental and numerical data, 0.89, was similar or greater than that reported in other studies [68,71,72,73,74].

Once the quasi-static modelling methodology was developed, calibrated and validated, the next step involved the adaptation of material property definitions to the dynamic loading scenario. Bone is widely described as a viscoelastic material. In other words, its material properties change with the loading rate. However, very few studies have taken this well-known fact into account, making this step extremely important [26,75].

Eight VB samples (n = 8) were prepared and tested; an impact load was applied via a falling mass, and surface displacements from the anterior surface of the bone were measured using DIC. The advent of non-contact measurement techniques, such as digital image correlation (DIC) and high-speed motion capture camera systems, enabled the supplementation of experimental findings by providing information on injury mechanisms and the kinematics of impact at the moment of the event. The load was acquired via two load cells positioned at the cranial and caudal ends of the sample. Load-displacement curves were produced and stiffness was calculated. Geometrical and numerical models were created following the developed methodology and a new calibration factor was calculated to adjust the relationship between density and Young’s modulus. It was assumed that ${\bar{K}}_{GS_{static}}$ factor already took into account specimen variability, and therefore, the approach of finding a constant of proportionality α would simplify and combine the processes of calibration and validation. Besides, the use of a single coefficient to relate dynamic and static grey-scale calibration factors greatly simplifies the modelling methodology, reducing the number of experimental trials required for this phase and providing a clear pathway in relating quasi-static and dynamic modelling approaches. With α = 4.3, and thus $K_{GS_{dynamic}}$ = 0.14, average numerical stiffness was 2.3% larger than experimental, 50,794 $N\;{\mathrm{mm}}^{-1}$ and 49,677 $N\;{\mathrm{mm}}^{-1}$, respectively.

In terms of stiffness variability, the results presented no statistically significant difference to experimental data; the large majority of the differences between numerical and experimental data ranged from 1% to 40% and the average difference for the datasets was 2.3%. These differences were similar to the quasi-static results, where the average difference was 3.7%, varying between 2% and 40%. Nevertheless, the $R^{2}$ value for the correlation plot of the dynamic models was less than 0.60. This was primarily caused by the small sample size and does not reflect the accuracy of the modelling approach, as demonstrated by box and whisker and Bland–Altman plots which indicated that good agreement was achieved.

Contour plots of the displacements for the VB surfaces were also assessed in this study, from both experimental and numerical data. The majority of the numerical contour plots were in agreement with experimental findings, mainly in the areas of maximum displacement, which were mostly located at the junctions with the bone cement cap. Similar locations are reported by other studies using FE models [56,68,76,77]. In the quasi-static experiments, agreement was better in the calibration dataset compared to the validation one. The dynamic set also exhibited good agreement, and maximum displacement areas were generally similar and located at the junction between the VB and cranial specimen holder, as expected. On the other hand, some plots, such as seen in Figure 7b, presented slightly different displacement contour distributions.

The present study made use of linear elastic models for material properties. This characteristic caused some models to have excessively distorted elements, which prevented the models from solving up to 10 kN. As the materials stiffen up after the yielding point; the introduction of elastoplastic models could have avoided some of the element distortions and allowed the models to solve to higher levels of loading.

Nevertheless, the use of linear elastic materials can be justified in this study: while yielding or plasticity have been incorporated in some models to predict bone failure sites and fracture mechanisms [31,62,63,78,79], this requires the introduction of additional parameters, which also require calibration, adding another variable to the modelling process. Additionally, the viscoelastic behaviour of the bone makes its stiffness increase with the loading rate. As the loading rate seen in impact collisions is relatively high, it is expected that the stiffness of the bone will increase drastically. As a consequence, bone might not achieve the yielding point when subjected to that loading condition, making the use of yielding properties unnecessary. Finally, had the yield point been achieved and a fracture occurred, such regions would exhibit high strain levels, even when modelled as a linear elastic material. Therefore, the use of yielding in this study is not strictly necessary and would add additional numerical variables to be calibrated using the same experimental data, increasing the uncertainties in the modelling process.

The main advantage of the modelling approach developed here is represented by the simplicity of the implied relationship between quasi-static and dynamic material properties. This, in turn, allowed a robust material calibration for dynamic events based on a limited number of experimental samples. In this dataset a difference of just over 2% was recorded between the experimental and numerical stiffness.

The models generated in this study predicted regions of maximum displacement, which were comparable in terms of magnitude and location to experimental data obtained by DIC, and reproduced the structural stiffness observed experimentally.

## 5. Conclusions

A calibrated, validated and robust finite element methodology for modelling vertebral bodies subjected to both quasi-static and dynamic impact loading is presented. With this methodology, we made accurate, specimen-specific, finite element models of porcine vertebral bodies harvested from porcine samples, allowing the prediction of compressive stiffness and regions of maximum displacement. In this study DIC was extensively used for model validation purposes as it allows comparisons of experimental surfaces’ displacements to those predicted with numerical models. Experimental tests were conducted to define the mechanical behaviour of vertebral bodies subject to impact loading and the finite element models were extended to encompass this case. This required a novel approach to assign adequate material properties to the dynamic models: a factor was defined to allow an adjustment to the material properties defined for the quasi-static case to account for the stiffening of the bone due to the increased rate of loading. The work pipeline developed is easily translated to VBs originating from other species, including humans, and it is proposed it could be applied to the creation of whole cervical spine models in order to investigate the consequences of impact loading on longer spinal segments.

The main advantage afforded by the adoption this modelling framework is that, by exploiting the inherit simplicity of the linear relationship postulated between quasi-static and dynamic material properties, it is possible to achieve robust model validation based on a relatively small number of experimental trials.

## Figures and Tables

**Table 1:** The sample list for each experiment.

		Spine N°	Level	Total
**Quasi-static** **compression**	**Calibration**	1	C4 to C6	**14 vertebral** **bodies**
		2	C2 to C5	
		3	C2, C5 and C6	
		4	C2 and C4	
		12	C2 to C4	
	**Validation**	4	C5 to C7	**13 vertebral** **bodies**
		5	C7	
		6	C7	
		12	C5 to C7	
		13	C2 to C5 and C7	
**Dynamic** **compression**		7	C2, C3, C5 and C7	**8 vertebral** **bodies**
		8	C7	
		9	C7	
		10	C7	
		11	C7	
	**Total**	**13 Spines**		**35 vertebral** **bodies**

**Table 2:** Material properties for bone cement, cartilage and steel plate.

Structure	Material	Model	Elastic Parameter [MPa]	Poisson	Density [km m${}^{-3}$]	Ref
Plate	Steel	Isotropic	*E* = 200 × 10${}^{3}$	0.3	8000	[58]
Cement	PMMA	Isotropic	*E* = 1177	0.35	1200	[Preliminary experiment]
Cartilage	Cartilage	Hyperelastic Mooney-Rivlin	$C_{10}$ = 0.24 $C_{01}$ = −0.06	-	1100	[59]

## Supplementary Materials

The following are available online at https://www.mdpi.com/1996-1944/13/19/4262/s1.
