# Supplementary material for: A Novel Modelling Methodology Which Predicts the Structural Behaviour of Vertebral Bodies under Axial Impact Loading: A Finite Element and DIC Study
Source: Materials (Basel). 2020 Sep 24;13(19):4262. doi: 10.3390/ma13194262 (PMC7578961; doi:10.3390/ma13194262)
Supplement: Supplementary file 1 [file materials-13-04262-s001.pdf]

Article

A novel modelling methodology which predicts the structural behaviour of vertebral bodies under axial impact loading: a finite element and DIC study.

Bruno Agostinho Hernandez [1,‡], **Harinderjit Singh Gill [1,]** and **Sabina Gheduzzi [1,‡*]**

[1] Department of Mechanical Engineering, University of Bath Bath, BA2 7AY; bah33@bath.ac.uk (BAH), rg433@bath.ac.uk (HSG), enssg@bath.ac.uk (SG)

Version September 23, 2020 submitted to Materials

1. Supplementary Material

This section presents the remaining results for the calibration and validation models.

1.1. Quasi-Static Calibration Results - Load-Displacement curves

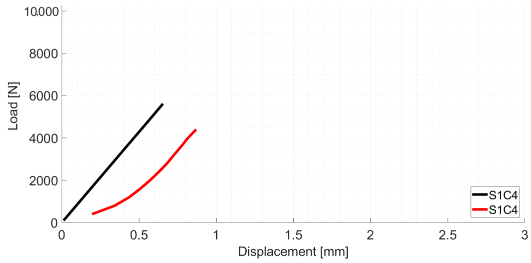

(a) Spine 01, Sample C4

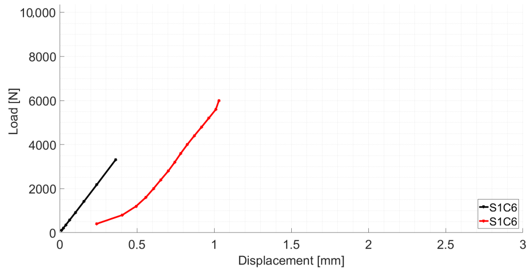

(b) Spine 01, Sample C6

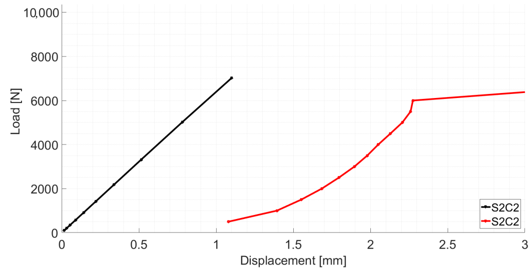

(c) Spine 02, Sample C2

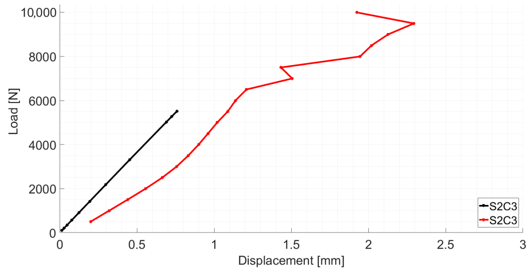

(d) Spine 02, Sample C3

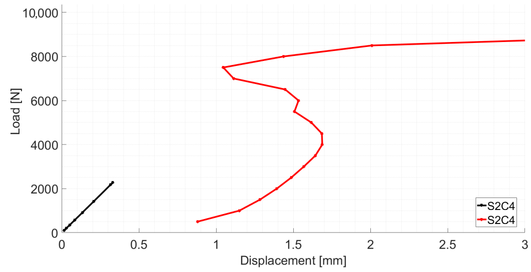

(e) Spine 02, Sample C4

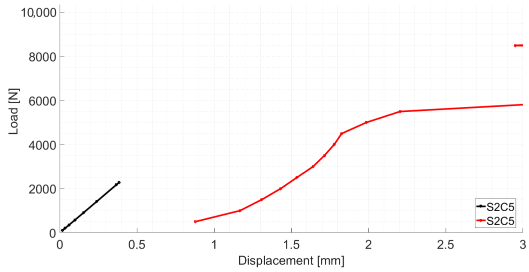

(f) Spine 02, Sample C5

Figure 1. Load-Displacement curves from the calibration models. Red curves are the DIC/experimental results. Black curves are the numerical results.

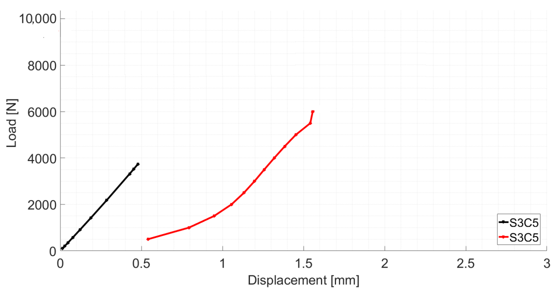

(a) Spine 03, Sample C5

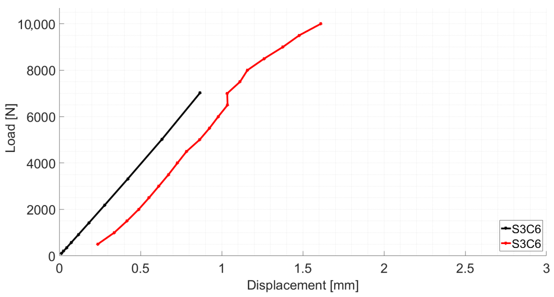

(b) Spine 03, Sample C6

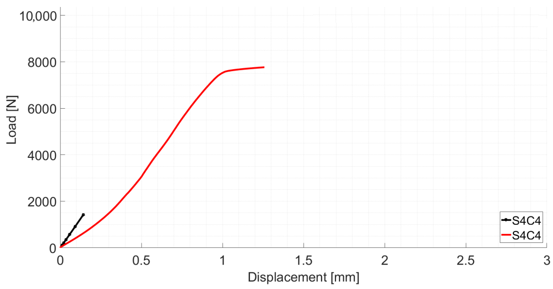

(c) Spine 04, Sample C4

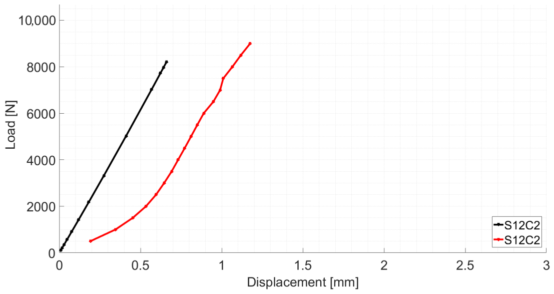

(d) Spine 12, Sample C2

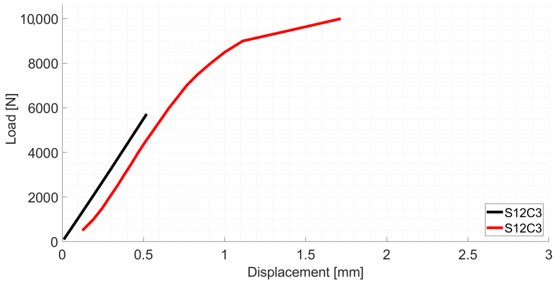

(e) Spine 12, Sample C3

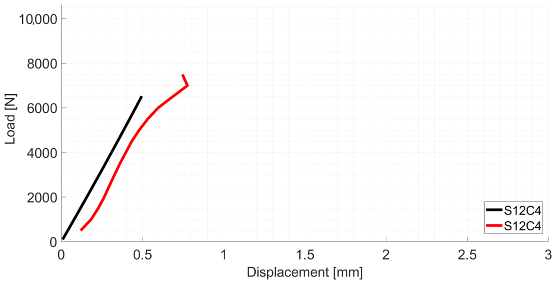

(f) Spine 12, Sample C4

Figure 2. Load-Displacement curves from the calibration models. Red curves are the DIC/experimental results. Black curves are the numerical results.

1.2. Quasi-Static Validation Results - Load-Displacement curves

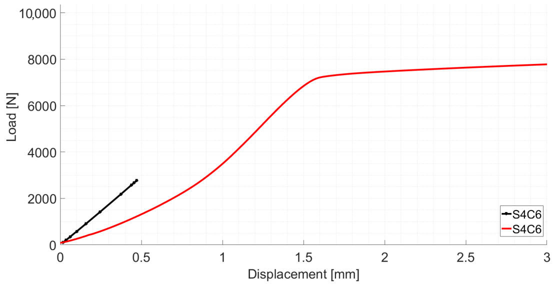

(a) Spine 04, Sample C6

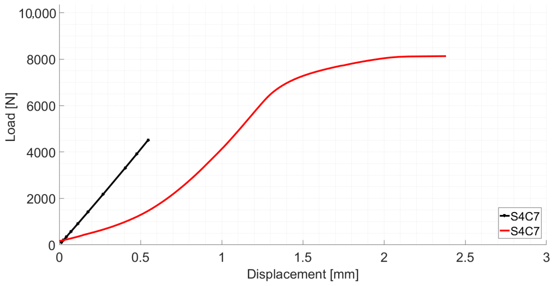

(b) Spine 04, Sample C7

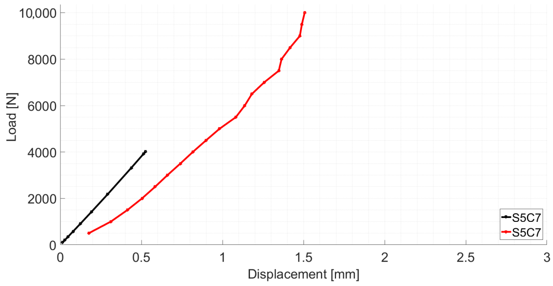

(c) Spine 05, Sample C7

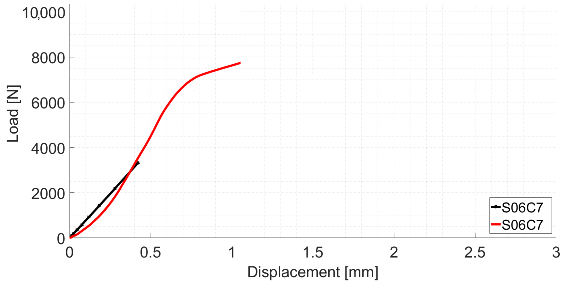

(d) Spine 06, Sample C7

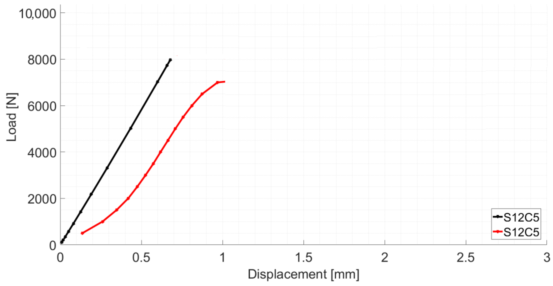

(e) Spine 12, Sample C5

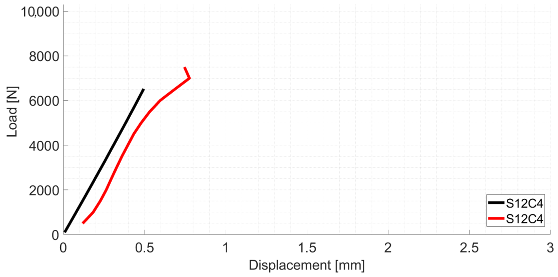

(f) Spine 12, Sample C6

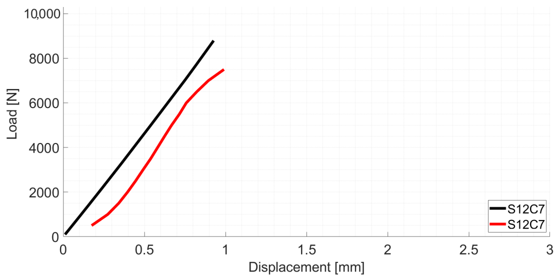

(g) Spine 12, Sample C7

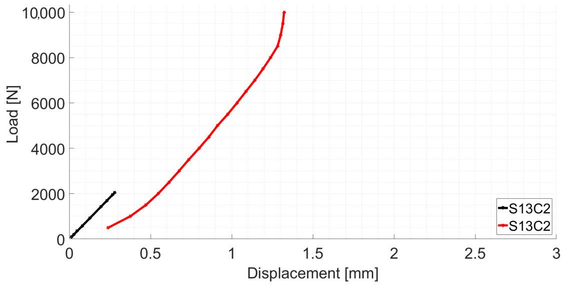

(h) Spine 13, Sample C2

Figure 3. Load-Displacement curves from the validation models. Red curves are the DIC/experimental results. Black curves are the numerical results.

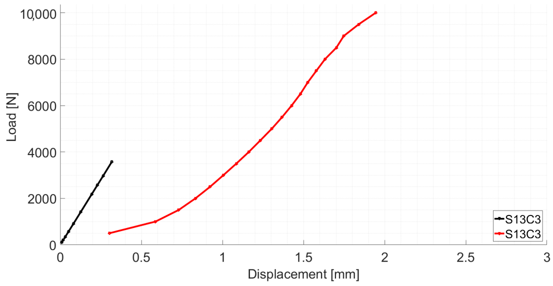

(a) Spine 13, Sample C3

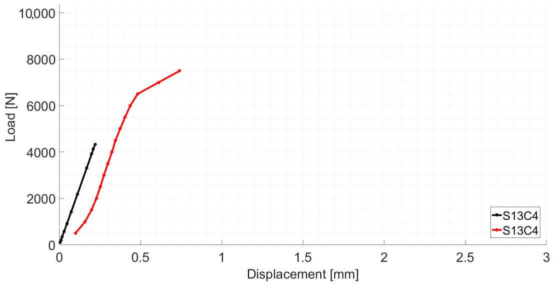

(b) Spine 13, Sample C4

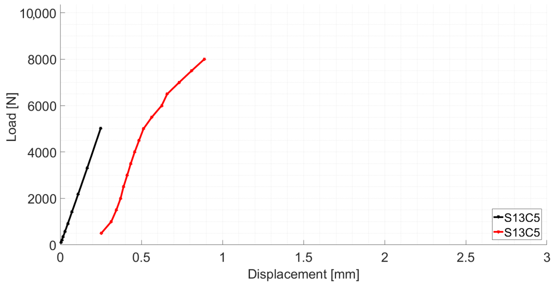

(c) Spine 13, Sample C5

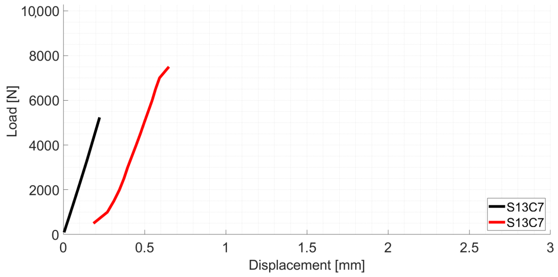

(d) Spine 13, Sample C7

Figure 4. Load-Displacement curves from the validation models. Red curves are the DIC/experimental results. Black curves are the numerical results.

1.3. Dynamic Results - Load-Displacement curves

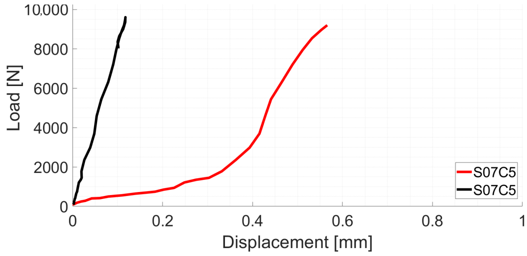

(a) Spine 07, Sample C5

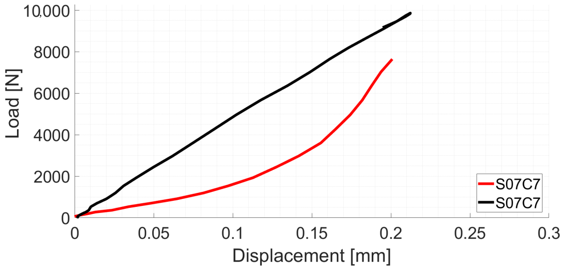

(b) Spine 07, Sample C7

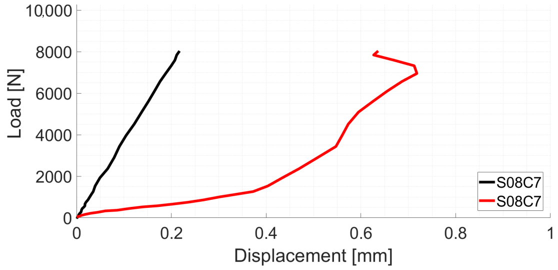

(c) Spine 08, Sample C7

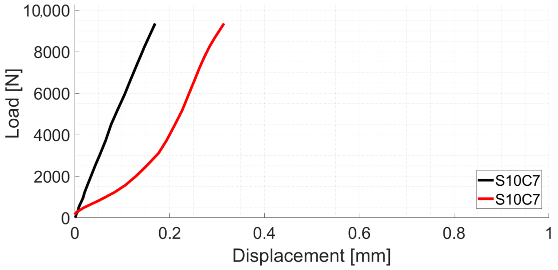

(d) Spine 10, Sample C7

Figure 5. Load-Displacement curves from the dynamic models. Red curves are the DIC/experimental results. Black curves are the numerical results.

© 2020 by the authors. Submitted to *Materials* for possible open access publication under the terms and conditions of the Creative Commons Attribution (CC BY) license (http://creativecommons.org/licenses/by/4.0/).
